# Improved diagnostic markers for invasive pulmonary aspergillosis in COPD patients

**DOI:** 10.3389/fcimb.2024.1294971

**Published:** 2024-04-03

**Authors:** Zhiwei Long, Xiaotong Li, Zhengtu Li, Jieying Hu, Ye Qiu, Shaoqiang Li, Yangqing Zhan, Feng Ye, Yan Wang

**Affiliations:** State Key Laboratory of Respiratory Disease, National Clinical Research Center for Respiratory Disease, Guangzhou Institute of Respiratory Health, The First Affiliated Hospital of Guangzhou Medical University, National Center for Respiratory Medicine, Guangzhou, China

**Keywords:** invasive pulmonary aspergillosis, chronic obstructive pulmonary disease, C-reactive protein, erythrocyte sedimentation rate, procalcitonin, lactate dehydrogenase, ceruloplasmin

## Abstract

**Background:**

The prevalence of invasive pulmonary aspergillosis (IPA) among patients with chronic obstructive pulmonary disease (COPD) is steadily increasing, leading to high mortality. Although early diagnosis can significantly reduce mortality, the efficacy of current diagnostic methods is limited. Consequently, there is a need for novel approaches for early IPA detection.

**Methods:**

This retrospective study involved 383 hospitalized COPD patients with GOLD stages III and IV. The IPA group (67 patients) and non-IPA group (316 patients) were identified at the First Affiliated Hospital of Guangzhou Medical University between January 2016 and February 2022. We analyzed common serological indicators in our hospital to identify predictive indicators for the early diagnosis of IPA in COPD patients.

**Results:**

The sensitivity and specificity of C-reactive protein (CRP), erythrocyte sedimentation rate (ESR), procalcitonin (PCT), lactate dehydrogenase (LDH), and ceruloplasmin (CER) for diagnosing IPA in COPD patients were as follows: CRP (91.2%, 57.7%), ESR (77.5%, 73.0%), PCT (60.5%, 71.4%), LDH (50.0%, 88.8%), and CER (60.7%, 74.3%). Combinations of biomarkers, such as CRP-ESR, CRP-LDH, ESR-LDH, ESR-CER, and LDH-CER, showed promising diagnostic potential, with larger area under the curve (AUC) values for IPA diagnosis in COPD patients. However, no statistically significant difference was observed between the diagnostic efficacy of single biomarkers and combined biomarkers. Notably, compared to those in the unassisted ventilation group, the patients in the assisted ventilation group (including noninvasive ventilation and tracheal intubation/incision-assisted ventilation group) exhibited significantly greater PCT and LDH levels, while the CER significantly decreased (p=0.021). There were no significant differences in biomarker levels between the ICU group and the non-ICU group. CRP (p<0.01), ESR (p=0.028), PCT (p<0.01), and CER (p<0.01) were positively correlated with hospitalization duration, whereas LDH was not correlated with hospitalization duration.

**Conclusion:**

Our study highlights the diagnostic potential of CRP, ESR, PCT, LDH, and CER for IPA in COPD patients. CRP and LDH can also initially predict the need for assisted ventilation, while CRP can initially estimate the length of hospitalization. This study represents the first report of the potential of CER for diagnosing IPA, suggesting its significance for further research.

## Introduction

Invasive pulmonary aspergillosis (IPA) is an opportunistic infectious disease that poses a significant threat to immunocompromised patients, leading to severe infections and fatalities. While previous research has associated IPA primarily with conditions such as blood disorders, organ transplants, agranulocytosis, and immunosuppression, emerging evidence indicates a rising incidence of IPA in patients with chronic obstructive pulmonary disease (COPD). Notably, recent studies have reported IPA occurrence rates ranging from 1.3% to 16.5% in the context of COPD, with up to 22.1% of COPD patients experiencing IPA infections (Guinea et al., 2010; Fortún et al., 2016; Gu et al., 2021). The mortality rates associated with combined COPD and IPA can reach 58.3% to 77.0% (Bulpa et al., 2007; Barberan et al., 2012; Delsuc et al., 2015). This high mortality is attributed to challenges in early diagnosis and treatment delay. Early detection of IPA in COPD patients is particularly challenging. In contrast to IPA in immunosuppressed patients, the clinical manifestations of IPA in COPD patients are often overshadowed by underlying conditions. Furthermore, the distinctive “air crescent sign” and “halo sign”, which are characteristic imaging indicators of IPA, are infrequently observed in COPD patients (Dai et al., 2013). Although lung tissue biopsy remains the gold standard for IPA diagnosis, its feasibility is hampered by the compromised lung function of COPD patients. Compared with that of immunosuppressed individuals, the utility of the galactomannan (GM) test—a commonly used diagnostic tool—among COPD patients is hindered by a lower positivity rate due to greater neutrophil activity capable of engulfing galactomannan (Cordonnier et al., 2009). Moreover, false-positives in the GM test pose significant challenges (Maertens et al., 2004; Guinea et al., 2010). Consequently, innovative methods are required to facilitate IPA diagnosis in COPD patients. Notably, commonly available serum biomarkers—C-reactive protein (CRP), erythrocyte sedimentation rate (ESR), procalcitonin (PCT), lactate dehydrogenase (LDH), and ceruloplasmin (CER)—have several advantages, including ease of detection, affordability, and noninvasiveness.

C-reactive protein (CRP), which is predominantly synthesized in the liver, is an acute-phase inflammatory protein composed of homopentamers (Sproston and Ashworth, 2018). Elevated CRP levels are commonly observed in patients with autoimmune diseases, infections, and certain cardiovascular conditions (Du Clos and Mold, 2004). Roques et al. demonstrated a significant increase in CRP in IPA patients with leukemia and neutropenia (Roques et al., 2016). Additionally, high CRP levels are associated with chronic pulmonary aspergillosis and poor prognosis (Hou et al., 2017; Phoompoung and Chayakulkeeree, 2020). Nonetheless, the influence of CRP on *Aspergillus* infections in COPD patients remains unexplored. The erythrocyte sedimentation rate (ESR), another widely used acute-phase indicator, plays a pivotal role in diagnosing and monitoring orthopedic infections (Barrack et al., 2019). While the ESR generally increases in response to infections as a nonspecific inflammatory marker, it has been proven useful in diagnosing IPA among immunocompromised patients (Badiee et al., 2022). Tong et al. reported a correlation between elevated ESR and poor IPA prognosis (Tong et al., 2021). Procalcitonin, which is predominantly secreted by human thyroid C cells at serum concentrations less than 0.05 ng/mL in healthy individuals (Xu et al., 2022), aids in distinguishing bacterial from nonbacterial infections and diagnosing sepsis (Gai et al., 2018; Downes et al., 2020; Pierrakos et al., 2020). Although the potential of PCT for diagnosing invasive fungal diseases has been recognized (Dou et al., 2013), its role in invasive aspergillosis remains underexplored and marked by limited and conflicting reports. Lactate dehydrogenase (LDH), a crucial enzyme in anaerobic glycolysis, catalyzes the conversion of lactic acid to pyruvate (Alam et al., 2018). LDH serves as a biomarker for various malignancies and malaria (Zhou et al., 2022). LDH levels are closely linked to *Aspergillus* virulence and tissue damage during *Aspergillus* infections (Zhai et al., 2021). Ceruloplasmin (CER), a serum ferrous oxidase that contains more than 95% of plasma copper, is an acute-phase protein that increases in response to inflammation and infection (Hellman and Gitlin, 2002). During fungal infections, the body elevates copper levels to counteract fungal cells and enhance macrophage phagocytosis (Besold et al., 2016). Goetting et al. documented increased CER levels in *Aspergillus*-infected birds, correlating high CER levels with poor prognosis (Goetting et al., 2013). Despite these insights, limited information exists regarding the role of CER in *Aspergillus* infections and its potential as a reliable serum biomarker. Previously, Badiee et al. demonstrated the utility of CRP, ESR, PCT, and LDH for the supportive biomarkers of invasive aspergillosis in immunocompromised patients, the vast majority of whom have hematologic disorders (Badiee et al., 2022). In contrast, our study population focused on nonimmunocompromised COPD patients. Moreover, Badiee et al.’s study focused on systemic invasive aspergillosis (e.g., in the lungs, blood, and kidneys), and our study was limited to invasive pulmonary aspergillosis. In addition, given the complex relationship between CER and Aspergillus, we also included CER in our study. It is unclear whether these biomarkers can be used as supportive biomarkers to aid in the diagnosis of IPA in non-immunocompromised COPD patients.

In conclusion, the literature on the relationship between biomarkers and invasive aspergillosis remains scarce, and related studies have focused primarily on immunosuppressed patients with blood disorders (Ambasta et al., 2015). Currently, no literature has examined the diagnostic value of CRP, ESR, PCT, LDH, and CER for IPA in the context of COPD. This study aimed to bridge this gap by investigating biomarker differences between COPD patients with and without IPA, providing fresh insights into early IPA diagnosis in COPD patients. Moreover, considering that the prediction of disease severity is also a part of early diagnosis, we explored the relationship between each biomarker and the need for assisted ventilation (including noninvasive ventilation and endotracheal intubation/incision-assisted ventilation), ICU admission, and length of hospitalization among COPD-IPA patients. The goal of this study was to identify severity predictors for IPA in COPD patients, enabling early intervention and improved patient outcomes.

## Materials and methods

### Study design

We conducted a retrospective analysis of the serum data of 67 hospitalized COPD-IPA patients at stages GOLD III and IV (IPA group) and 316 hospitalized COPD patients at stages GOLD III and IV (non-IPA group) from the First Affiliated Hospital of Guangzhou Medical University between January 2016 and February 2022. COPD diagnosis and GOLD grading were performed according to the Global Initiative for Chronic Obstructive Lung Disease (GOLD) guidelines (Agustí et al., 2023). In addition, other patient information, including demographic data (sex, age, smoking status, BMI), clinical manifestations, radiographic findings, microbiological test results, laboratory data (white blood cell count, blood gas analysis, CRP, ESR, PCT, LDH, CER), length of hospitalization, ICU admission, assisted ventilation, and indwelling catheter use, was obtained from electronic records. The IPA group was matched with the non-IPA group in terms of GOLD classification, age, and sex.

### Patient inclusion

Inclusion of the IPA group: 1. Meeting the “proven invasive aspergillosis” or “probable invasive aspergillosis” criteria in the Bulpa criteria (Bulpa et al., 2007) (diagnostic criteria provided in the **Appendix**); 2. The following conditions cannot be met: 1). Malignant tumors, blood diseases; 2). Severe congenital immunodeficiency; 3). Connective tissue disease; 4). Treatment with corticosteroids, averaging a minimum dose of 2 mg/kg/day prednisone equivalent for >2 weeks; 5). Other severe immunosuppressive states. The inclusion criteria for the non-IPA group were as follows: 1. No evidence of *Aspergillus* infection; 2. The same exclusion criteria for the experimental group were applied to the control group.

### Biomarker collection and detection

All biomarkers were assessed at admission before the patients received antifungal medication. CRP was assessed using the turbidimetric inhibition immunoassay method (C-reactive protein kit, Beckman Coulter). The ESR was measured using capillary photometer imaging (ALIFAX Automated Erythrocyte Sedimentation Rate Analyzer TEST1/THI, Shanghai Jumo Medical Equipment Co.). PCT was detected using enzyme-linked immunofluorescence (Procalcitonin Kit, BioMerieux). LDH was quantified using the rate method (Lactate Dehydrogenase Kit, Beckman Coulter). The CER was evaluated through the turbidimetric inhibition immunoassay method (Ceruloplasmin Kit, Beckman Coulter).

### Statistical analysis

We used SPSS version 25.0 (IBM Corp, Armonk, NY, USA) for the statistical analysis. The normality tests included the Kolmogorov−Smirnov and Shapiro−Wilk tests. Continuous variables are presented as medians and quartile ranges. The t test or Mann−Whitney test was used for continuous variable comparisons, while the chi−square test or Fisher’s exact test was used for categorical variable comparisons. Univariate Cox hazard analysis was used to test for differences in biomarkers between patients admitted to the ICU and non-ICU patients within the IPA group. Spearman correlation analysis was used for correlation analysis. GraphPad Prism version 9.5.1 (GraphPad Software, Boston, Massachusetts, USA) was utilized to analyze the sensitivity, specificity, cutoff values, and ROC curves for each biomarker. MedCalc statistical software version 20.022 (MedCalc Software bv, Ostend, Belgium) was used to calculate positive predictive values, negative predictive values, and test differences between ROC curves. Statistical significance was set at p<0.05.

## Results

### Patient characteristics

A total of 383 hospitalized COPD patients at stages III and IV were included in the study. Among them, 67 patients were classified into the IPA group, while 316 patients formed the control group. The median age of the IPA group was 67 years, with 60 (89.6%) males and 7 (10.4%) females. The median age of the non-IPA group was 68 years, with 288 (91.1%) males and 28 (8.9%) females. Smoking status and BMI did not significantly differ between the two groups. In terms of clinical symptoms, the IPA group displayed significantly greater proportions of fever (64.0% vs. 18.1%, p<0.01) and hemoptysis (43.2% vs. 12.0%, p<0.01), while dyspnea was significantly less common (88.5% vs. 97.0%, p=0.018). No significant differences were observed in cough or chest pain between the two groups. Radiographic findings revealed significantly more infiltrates (47.8% vs. 19.0%, p<0.01), cavities (19.4% vs. 5.1%, p<0.01), and pleural effusion (25.4% vs. 5.1%, p<0.01) in the IPA group. Notably, typical IPA signs were rare in the IPA group, with only 1.5% each for the halo sign and air crescent sign. White blood cell count (median 11.5 x 10^9 vs. 7.9 x 10^9, p<0.01) and neutrophil count (median 9.1 x 10^9 vs. 5.5 x 10^9, p<0.01) were significantly greater in the IPA group, while lymphocyte count was significantly lower (median 0.8 x 10^9 vs. 1.5 x 10^9, p<0.01). Blood gas analysis indicated significantly greater actual HCO3- levels in the IPA group (median 27.2 mmol/L vs. 26.0 mmol/L, p=0.042), with no significant differences in pH, PaCO2, or PaO2 between the two groups. Significant differences between the IPA and non-IPA groups were observed in noninvasive ventilator-assisted ventilation (65.7% vs. 21.2%, p<0.01), endotracheal intubation or incision-assisted ventilation (41.8% vs. 13.7%, p<0.01), indwelling catheter use (68.7% vs. 30.1%, p<0.01), ICU admissions (37.3% vs. 4.1%, p<0.01), and length of hospitalization (median 14 vs. 6 days, p<0.01). The baseline patient data are summarized in **Table 1**.

**Table 1:** Baseline data for patients in the IPA and non-IPA groups^a^.

	IPA group (n=67)	non-IPA group (n=316)	*p* value^b^
Demographic data			
Age	67.0 (61.0~71.0)	68.0 (62.3~76.0)	0.313
Male gender	60 (89.6%)	288 (91.1%)	0.682
Current smokers*	20/55 (36.4%)	94/238 (39.5%)	0.668
BMI	21.1 (18.3~23.5)	20.2 (17.8~22.0)	0.124
Clinical symptoms			
Fever*	32/50 (64%)	41/226 (18.1%)	<0.01
Cough*	55/56 (98.2%)	243/267 (91.0%)	0.119
Chest pain*	6/39 (15.4%)	28/210 (13.3%)	0.732
Dyspnea*	46/52 (88.5%)	257/265 (97.0%)	0.018
Hemoptysis*	16/37 (43.2%)	21/175 (12.0%)	<0.01
Radiography findings			
Infiltrates	32 (47.8%)	60 (19.0%)	<0.01
Cavity	13 (19.4%)	16 (5.1%)	<0.01
Nodules	2 (3.0%)	6 (1.9%)	0.925
Halo sign	1 (1.5%)	0 (0)	0.392
Air crescent sign	1 (1.5%)	0 (0)	0.392
Pleural effusion	17 (25.4%)	16 (5.1%)	<0.01
White blood cell count (×10^9^)			
White blood cell	11.5 (8.1~18.2)	7.9 (6.4~9.7)	<0.01
Neutrophils	9.1 (6.3~16.0)	5.5 (3.7~7.2)	<0.01
Lymphocyte	0.8 (0.50~1.30)	1.50 (1.00~2.00)	<0.01
Blood gas analysis			
PH	7.4 (7.36~7.44)	7.4 (7.37~7.42)	0.165
PaCO_2_	45.0 (37.1~52.9)	43.6 (39.5~47.8)	0.551
PaO_2_	87.2 (73.3~98.7)	94.0 (81.5~99.1)	0.094
Actual bicarbonate (mmol/L)	27.2 (24.2~30.3)	26.0 (23.5~28.4)	0.042
Respiratory support			
Noninvasive ventilator-assisted ventilation	44 (65.7%)	67 (21.2%)	<0.01
Endotracheal intubation/Incision assisted ventilation	28 (41.8%)	38 (13.7%)	<0.01
Indwelling catheters	46 (68.7%)	95 (30.1%)	<0.01
Hospitalization (Day)	14 (8~22)	6 (4~9)	<0.01
ICU admission	25 (37.3%)	13 (4.1%)	<0.01

IPA, invasive pulmonary aspergillosis; BMI, body mass index; ICU, intensive care unit.

^a^Data are presented as the median (interquartile range) or no. (%).

^b^p < 0.05 was considered to indicate statistical significance.

*Data for some patients are missing, and the total numbers included in the calculation are recorded in a table.

### Biomarkers for IPA diagnosis in COPD patients

**Figure 1:**
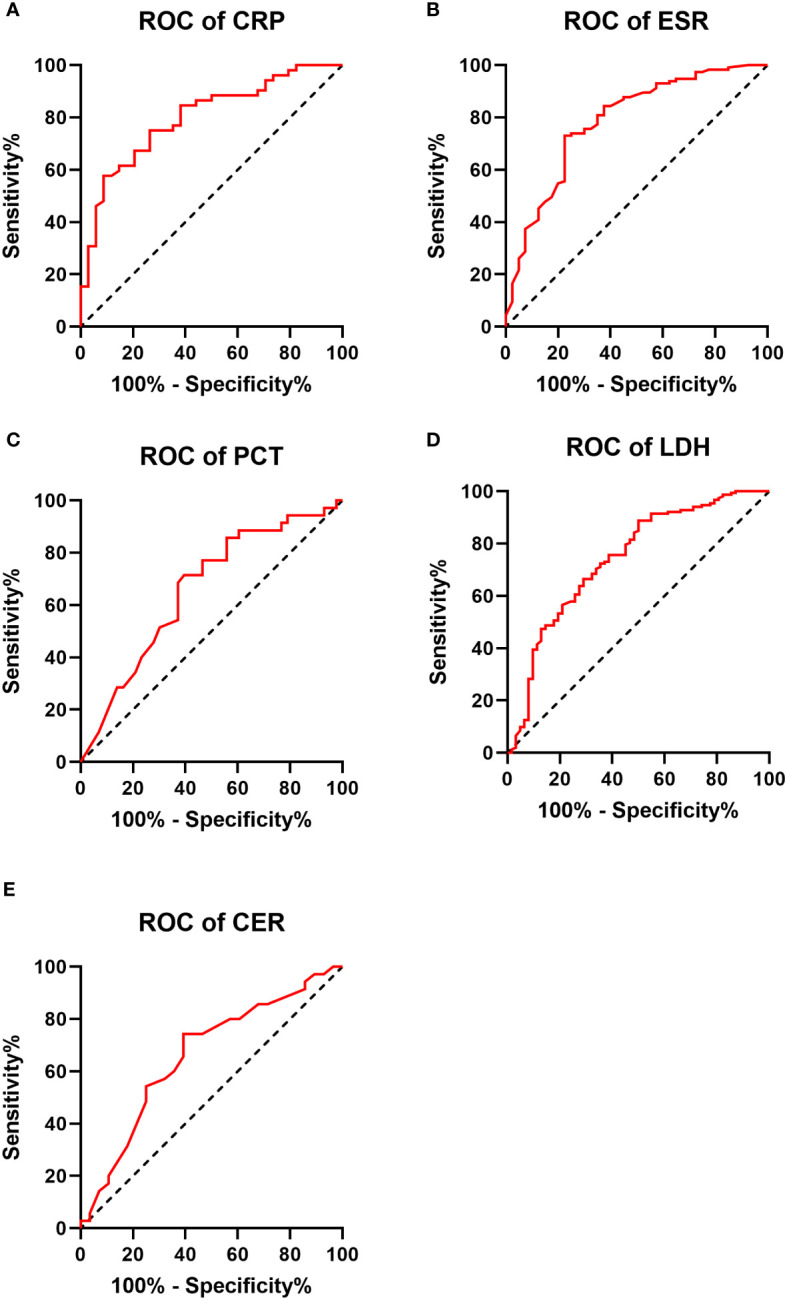
Receiver operating characteristic (ROC) curve for each biomarker in the IPA and non-IPA groups **(A–E)**.

Significant differences were observed between the IPA and non-IPA groups for CRP (median 10.49 mg/L vs. 1.08 mg/L, p<0.01), ESR (median 69.0 mm/h vs. 20.0 mm/h, p<0.01), PCT (median 0.29 ng/mL vs. 0.12 ng/mL, p=0.016), LDH (median 234.1 U/L vs. 173.7 U/L, p<0.01), and CER (median 0.35 g/L vs. 0.28 g/L, p=0.023). **Table 2** presents these differences in detail. ROC curves were generated to evaluate the diagnostic performance of each biomarker for IPA in COPD patients (**Figure 1**). The area under the curve (AUC), cutoff values, sensitivity, specificity, positive predictive value (PPV), and negative predictive value (NPV) for each biomarker are summarized in **Table 3**. The AUC values for CRP, ESR, PCT, LDH, and CER were 0.799, 0.786, 0.659, 0.743, and 0.661, respectively. The corresponding cutoff values were 1.29, 31, 0.165, 239, and 0.33 g/L, respectively. The sensitivity and specificity were as follows: CRP (10.6%, 99.2%), ESR (13.6%, 98.3%), PCT (10.4%, 97.1%), LDH (19.7%, 97.0%), and CER (11.5%, 97.2%).

**Table 2:** Biomarker data for patients with and without IPA^a^.

	IPA group	non-IPA group	*p* value^b^
C-reactive protein(mg/L)	10.49(45.89, 2.82~48.7)	1.08(3.74, 0.29~4.03)	<0.01
Erythrocyte sedimentation rate(mm/h)	69.0(72.0, 34.3~106.5)	20.0(31.0, 9~40)	<0.01
Procalcitonin(ng/mL)	0.29(0.69, 0.11~0.80)	0.12(0.18, 0.07~0.25)	0.016
Lactate dehydrogenase(U/L)	234.1(111.6, 181.4~293.0)	173.7(63.7, 144.9~208.7)	<0.01
Ceruloplasmin(g/L)	0.35(0.12, 0.28~0.40)	0.28(0.1, 0.25~0.35)	0.023

^a^Data are presented as the median (interquartile range).

^b^p < 0.05 was considered to indicate statistical significance.

**Table 3:** Area under the curve (AUC), cutoff value, sensitivity, specificity, positive predictive value (PPV), and negative predictive value (NPV) for the diagnosis of COPD combined with IPA by each biomarker.

	AUC	Cutoff	Sensitivity	Specificity	PPV	NPV
C-reactive protein (mg/L)	0.799	1.29	91.2%	57.7%	14.7%	98.8%
Erythrocyte sedimentation rate (mm/h)	0.786	31	77.5%	73.0%	18.7%	97.6%
Procalcitonin (ng/mL)	0.659	0.165	60.5%	71.4%	14.5%	95.8%
Lactate dehydrogenase (U/L)	0.743	239	50.0%	88.8%	26.3%	95.7%
Ceruloplasmin (g/L)	0.661	0.33	60.7%	74.3%	15.9%	95.9%

### Combination of biomarkers

**Figure 2:**
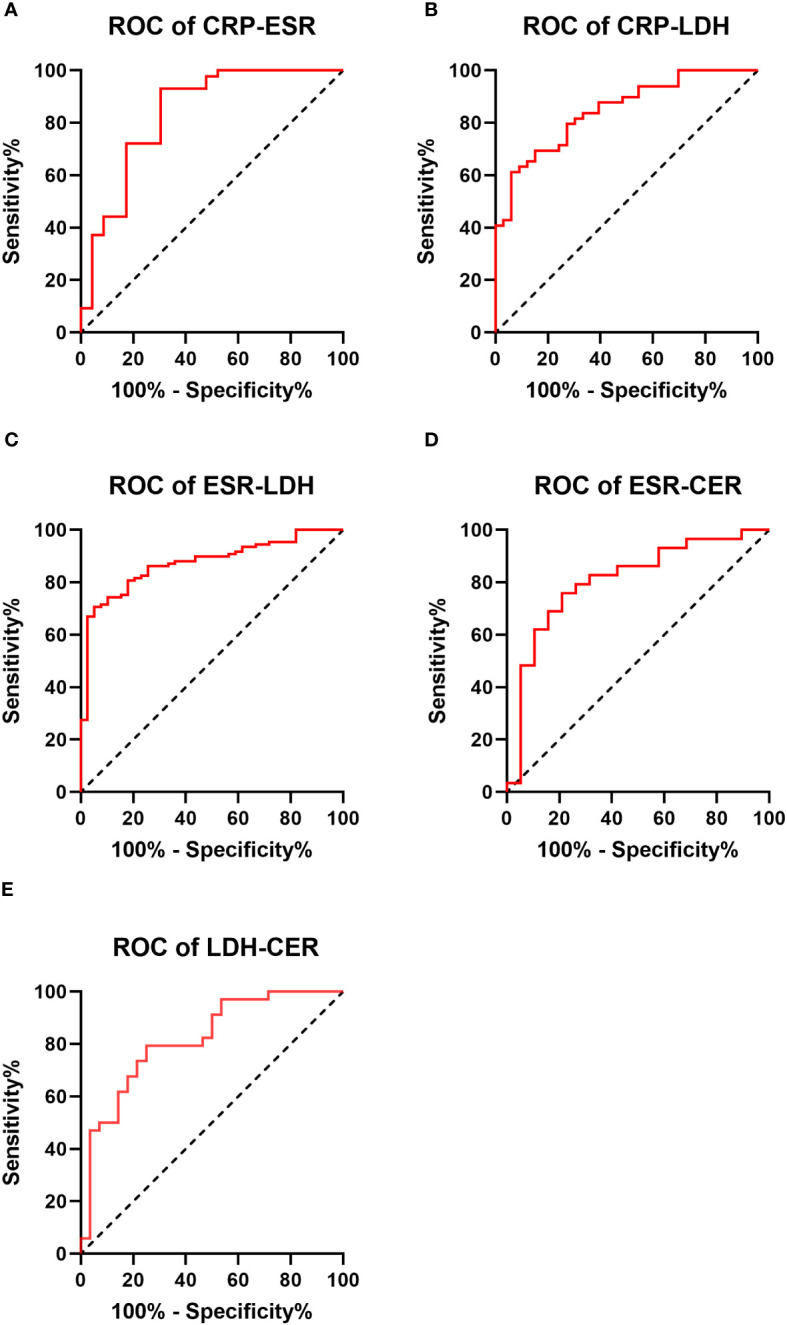
Receiver operating characteristic curve (ROC curve) for 2 biomarkers combined in the IPA and non-IPA groups (those with a larger area under the curve than a single biomarker) **(A–E)**.

ROC curves depicting combinations of two biomarkers are displayed in **Figure 2** (those with a larger area under the curve than a single biomarker). Those with a smaller area under the curve than that of a single biomarker are recorded in the **Appendix**. The AUC, cutoff values, sensitivity, specificity, PPV, and NPV for these combinations are outlined in **Table 4**. The AUC values for the CRP-ESR, CRP-LDH, ESR-LDH, ESR-CER, and LDH-CER were greater than those for the individual biomarkers (0.835, 0.847, 0.873, 0.808, and 0.818, respectively). However, only the difference between the ESR-LDH and ESR was statistically significant (p=0.045). Further details regarding the differences in ROC curves between single biomarkers and combinations are summarized in **Table 5** and the **Appendix**.

**Table 4:** Area under the curve (AUC), sensitivity, specificity, positive predictive value (PPV), and negative predictive value (NPV) for the diagnosis of COPD combined with IPA by two biomarker combinations.

	AUC	Sensitivity	Specificity	PPV	NPV
CRP-ESR	0.835	69.6%	93.0%	44.3%	97.5%
CRP-PCT	0.747	92.0%	58.3%	15.0%	98.9%
CRP-LDH	0.847	93.9%	61.2%	16.2%	99.2%
CRP-CER	0.775	81.8%	68.0%	17.0%	97.9%
ESR-PCT	0.708	63.3%	83.3%	23.3%	96.6%
ESR-LDH	0.873	94.9%	70.6%	20.5%	99.4%
ESR-CER	0.808	78.9%	75.9%	20.8%	97.8%
PCT-LDH	0.709	58.1%	87.5%	27.1%	96.3%
PCT-CER	0.654	68.4%	71.4%	16.1%	96.6%
LDH-CER	0.818	79.4%	75.0%	20.3%	97.8%

CRP, C-reactive protein; ESR, erythrocyte sedimentation rate; PCT, procalcitonin; LDH, lactate dehydrogenase; CER, ceruloplasmin.

**Table 5:** P value of the difference in the receiver operating characteristic (ROC) curve for the diagnosis of IPA in patients with COPD by one biomarker and two biomarkers.

	CRP	ESR	PCT	LDH	CER
CRP-ESR	0.337	0.480	0.958	0.325	0.542
CRP-PCT	0.428	0.946	0.901	0.299	0.503
CRP-LDH	0.088	0.154	0.204	0.371	0.254
CRP-CER	0.628	0.700	0.674	0.172	0.175
ESR-PCT	0.499	0.480	0.959	0.252	0.374
ESR-LDH	0.080	**0.045**	0.229	0.234	0.362
ESR-CER	0.245	0.210	0.658	0.895	0.647
PCT-LDH	0.136	0.392	0.532	1.000	0.947
PCT-CER	0.183	0.162	0.416	0.778	0.399
LDH-CER	0.494	0.051	0.127	0.135	0.177

p < 0.05 was considered to indicate statistical significance.

Values less than 0.05 are marked in bold.

CRP, C-reactive protein; ESR, Erythrocyte sedimentation rate; PCT, Procalcitonin; LDH, Lactate dehydrogenase; CER, Ceruloplasmin.

### Biomarkers and assisted ventilation in IPA-COPD patients

Within the IPA group, 44 patients received ventilator-assisted ventilation (including noninvasive and endotracheal intubation/incision-assisted ventilation), while 23 did not. PCT (median 0.55 vs. 0.13, p=0.030), LDH (median 251.5 vs. 197.9, p=0.024), and CER (median 0.33 vs. 0.38, p=0.021) were significantly greater in the ventilated group than in the nonventilated group. ESR and CRP did not exhibit significant differences. Biomarker data for the ventilated and nonventilated groups can be found in **Table 6**.

**Table 6:** Biomarker data in patients with and without assisted ventilation in the IPA group^a^.

	Assisted ventilation group (n=44)	non-Assisted ventilation group (n=23)	*p* value^b^
C-reactive protein (mg/L)	6.0 (2.4~45.1)	15.2 (3.9~70.2)	0.632
Erythrocyte sedimentation rate (mm/h)	47.0 (19.5~100.0)	87.0 (54.0~110.0)	0.083
Procalcitonin (ng/mL)	0.55 (0.13~1.68)	0.13 (0.08~0.38)	0.030
Lactate dehydrogenase (U/L)	251.5 (196.8~353.9)	197.9 (173.5~254.0)	0.024
Ceruloplasmin (g/L)	0.33 (0.25~0.40)	0.38 (0.35~0.45)	0.021

^a^Data are presented as the median (interquartile range).

^b^p < 0.05 was considered to indicate statistical significance.

### Biomarkers and ICU admission in IPA-COPD patients

In the IPA group, 25 patients were admitted to the ICU, while 42 were not. No significant differences were observed in CRP, ESR, PCT, LDH, or CER between patients admitted to the ICU and those who were not. **Table 7** shows the results of the univariate Cox hazard analysis of biomarker differences between patients admitted to the ICU and non-ICU patients in the IPA group.

**Table 7:** Univariate Cox hazard analysis of differences in biomarkers between patients admitted to the ICU and non-ICU patients in the IPA group.

	HR	95%CI	*p* value^a^
C-reactive protein(mg/L)	1.002	0.992-1.013	0.689
Erythrocyte sedimentation rate(mm/h)	0.995	0.976-1.013	0.562
Procalcitonin(ng/mL)	0.979	0.935-1.025	0.307
Lactate dehydrogenase(U/L)	1.002	0.999-1.006	0.259
Ceruloplasmin(g/L)	0.378	0-1023.828	0.810

^a^p < 0.05 was considered to indicate statistical significance.

### Biomarkers and hospitalization in IPA-COPD patients

The median hospitalization length for the IPA group was 14 days (interquartile range: 8~22 days). CRP, ESR, PCT, and CER were positively correlated with hospitalization length (p values <0.01, 0.028, <0.01, and <0.01, respectively), with correlation coefficients of 0.473, 0.249, 0.272, and 0.301, respectively. LDH exhibited no significant correlation. The detailed results of the Spearman correlation analysis are presented in **Table 8**.

**Table 8:** Association of biomarkers with length of hospital stay in patients with IPA.

	r	*p* value^a^
C-reactive protein	0.473	<0.01
Erythrocyte sedimentation rate	0.249	0.028
Procalcitonin	0.272	<0.01
Lactate dehydrogenase	0.184	0.149
Ceruloplasmin	0.301	<0.01

^a^p < 0.05 was considered to indicate statistical significance.

## Discussion

Early diagnosis and timely treatment of invasive pulmonary aspergillosis (IPA) in COPD patients present considerable challenges. Our study represents the first report on the diagnostic and severity prediction roles of CRP, ESR, PCT, LDH, and CER in IPA among COPD patients. Notably, this study is the first to investigate CER as a supportive biomarker for IPA.

CER, which regulates copper and iron metabolism, is also an acute-phase reaction protein whose expression increases during inflammation and infection (Hellman and Gitlin, 2002). It plays a complex role in fungal infections. On the one hand, fungi, as eukaryotic organisms, require copper as a nutrient and use CER as a preferred source of copper uptake (Besold et al., 2021). On the other hand, the body actively increases serum copper levels through hepatic production of CER, and high levels of copper are toxic to fungal pathogens and enhance their ability to be phagocytosed by macrophages (Besold et al., 2016; Culbertson et al., 2020). Iron is another important nutrient required by fungi. Unlike elevated serum copper levels, in fungal infections, the body actively reduces serum iron levels and develops nutritional immunity to the pathogen (Weinberg, 1975). As a ferrous oxidase, CER regulates iron metabolism (Hellman and Gitlin, 2002) and contributes to iron nutritional immunity against fungal pathogens. Additionally, CER combats oxidation, scavenges free radicals, and protects against tissue damage caused by excessive inflammation (Yang et al., 1996). Elevated CER levels indicated disease recurrence in a Coccidioidium paracoccidioides patient follow-up study (Sylvestre et al., 2018). In an animal study, CER levels increased in *Aspergillus*-infected birds, with a higher CER predicting individual death (Goetting et al., 2013). In our study, CER levels were significantly greater in the IPA group than in the non-IPA group (p=0.023). The best cutoff for diagnosing IPA in COPD patients was >0.33 g/L, with a sensitivity of 60.7% and a specificity of 74.3%. Our study marks the first report on the role of the CER as a supportive biomarker for IPA, yet its diagnostic performance appears to be modest. In our study, the AUC of CER was lower than that of CRP, ESR, and LDH, suggesting that early changes in CER are not as pronounced as those of other biomarkers in nonimmunosuppressed COPD patients. Compared with that of the GM test, which is traditionally used to detect IPA, the ROC area of the CER may be greater or similar to that of the serum GM test but lower than that of the BALF GM test (Fortún et al., 2016). However, obtaining BALF requires invasive maneuvers that are difficult for many COPD patients to tolerate. The positive rate of serum GM tests is also not ideal (Aquino et al., 2012). We do not recommend CER as the preferred of choice for the supportive biomarker of nonimmunocompromised COPD-IPA patients. CER testing can be used as a supplement in the case of a negative serum GM test. Importantly, certain COPD patients undergo extensive corticosteroid treatment, resulting in immunosuppression. In IPA patients in this subgroup, the pathological status is associated with an excessive host response rather than with *Aspergillus* invasion (Berenguer et al., 1995; Balloy et al., 2005). It remains unclear whether serum CER decreases due to imbalanced immune responses or increases to counteract excessive damage by scavenging free radicals. Moreover, whether the diagnostic efficacy of CER is excellent in COPD patients with heavy corticosteroid usage requires further investigation.

A CRP level above 5 mg/L has demonstrated a sensitivity and specificity of 94.1% and 100%, respectively, in diagnosing invasive aspergillosis in immunosuppressed patients (Badiee et al., 2022). In our study, the median CRP level in COPD patients with IPA was 10.49 mg/L, with a diagnostic cutoff of >1.29 mg/L, resulting in a sensitivity of 91.2% and specificity of 57.7%. Comparatively, our cutoff value was lower, potentially due to the lower CRP levels in the IPA group. Consistent with our findings, a study focusing on IPA survival follow-up reported a median CRP level of 14.1 mg/L in survivors (Tong et al., 2018). Notably, our biomarker data were collected on the first day of hospitalization, suggesting that lower CRP levels could indicate well-controlled disease and a relatively early IPA stage. Because the increase in CRP is more pronounced in the acute phase than most other acute proteins, CRP is often the first concern for clinicians. Nevertheless, the increase in other acute reactive proteins should not be ignored. A reported cutoff above 0.26 ng/mL yielded a sensitivity and specificity of 71.0% and 100%, respectively, for PCT for diagnosing invasive aspergillosis among 52 immunocompromised patients (Badiee et al., 2022). Similarly, our study revealed a mild increase in PCT in the IPA among COPD patients, with a diagnostic cutoff of >0.165 ng/mL and a sensitivity and specificity of 60.5% and 71.4%, respectively. Notably, compared to bacterial infections, *Aspergillus* infection leads to significantly lower PCT increases (Marková et al., 2013), which is related to the immunoreaction of *Aspergillus* infections. In *Aspergillus* infections, a significant increase in IFN-γ levels activates monocytes against *Aspergillus* (Warris et al., 2005), concurrently inhibiting PCT secretion (Linscheid et al., 2003). Given the moderate PCT elevation during *Aspergillus* infection, its diagnostic utility is limited. However, when coupled with substantial increases in other inflammatory biomarkers, such as CRP and ESR, even slight PCT elevation plays a considerable diagnostic role in *Aspergillus* infection. In our study, the best cutoff value of LDH for diagnosing IPA in COPD patients was >239 U/L, while the ESR was >31 mm/h. These findings align with prior reports (Tong et al., 2021; Badiee et al., 2022). Our study indicated that the cutoff values of LDH and the ESR for diagnosing IPA do not differ well between COPD patients and immunocompromised patients.

Overall, our study demonstrated the diagnostic potential of all five biomarkers for IPA among COPD patients. According to the AUC values, CRP, ESR, LDH, CER, and PCT ranked from highest to lowest. Combinations such as CRP-ESR, CRP-LDH, ESR-CER, LDH-CER, and LDH-CER exhibited larger AUCs than single biomarkers, although significant differences were not detected. Combining two biomarkers does not appear to substantially enhance diagnostic performance.

We proceeded to evaluate whether serum biomarkers correlated with the severity of IPA in COPD patients. Our findings suggest that while CRP cannot predict the need for assisted ventilation or ICU admission, a moderate positive correlation exists between CRP levels and the length of hospitalization. In line with Chai et al., we observed that higher CRP levels were associated with poor response to treatment and increased mortality among IPA patients (Chai et al., 2010). This finding parallels our study, suggesting that elevated CRP levels correlate with IPA severity. We propose that CRP could serve as a biomarker for predicting hospitalization duration in COPD patients with IPA. A retrospective study of 117 IPA patients demonstrated higher ESR levels among nonsurvivors than among survivors (Tong et al., 2021). This finding supports the use of the ESR as a serum biomarker for predicting IPA severity. However, our study revealed that the ESR could not predict the need for assisted ventilation or ICU admission, and the correlation between the ESR and hospitalization length was weak. Therefore, the ESR might not be suitable for early-stage IPA severity prediction.

PCT levels substantially increase during bacterial infections, making PCT useful for the diagnosis and monitoring of bacterial infections and sepsis. In sepsis, elevated PCT levels in the early stages correlate with severity and prognosis throughout the disease course (Becker et al., 2010; Gai et al., 2018). Conversely, PCT exhibits a slight increase in fungal infections, but studies on its role are limited. In chronic pulmonary aspergillosis, the diagnostic and monitoring efficacy of PCT is modest (Sehgal et al., 2023). Our study linked elevated PCT levels to the need for assisted ventilation and extended hospital stays. However, given the limited PCT elevation in *Aspergillus* infection, PCT alone might not effectively predict IPA severity. Combining PCT with other biomarkers appears promising for distinguishing between bacterial and fungal infections.

During fungal infections, LDH levels are correlated with fungal pathogen virulence and associated tissue damage (Zhai et al., 2021). In nonneutropenic IPA patients, serum LDH levels above 220 U/L were a risk factor for death (Dai et al., 2013). Our study established a connection between increased LDH levels and IPA severity in COPD patients, with high serum LDH indicating the need for assisted ventilation. Therefore, early respiratory support is crucial for improving the prognosis of COPD-IPA patients with high LDH levels. Sylvestre et al. reported that elevated CER levels indicate disease recurrence in Coccidioidium paracoccidioidium patients, with levels decreasing posttreatment (Sylvestre et al., 2018). Similarly, CER levels in deceased *Aspergillus*-infected birds exceeded those in survivors, with a positive correlation between CER levels and *Aspergillus* burden (Goetting et al., 2013). Although the role of CER in COPD-associated IPA remains unclear, previous reports have suggested an association between CER and disease severity. Intriguingly, our study revealed lower CERs in the assisted ventilation group than in the nonventilated ventilation group, with a positive correlation between CERs and hospitalization length. The incongruity in CER levels between assisted ventilation and hospitalization length remains unexplained. The role of the CER in determining COPD-IPA severity requires further investigation.

In conclusion, early respiratory support initiation and robust therapies are recommended for COPD-IPA patients with high CRP and LDH levels to improve prognosis. However, the role of the CER as a severity predictor in COPD-IPA patients remains inconclusive and requires further research.

Bacterial-fungal co-infection is a significant topic. Co-infections could make the patient’s condition more complicated and pose significant diagnostic and therapeutic challenges. It is widely believed that patients with compromised immune systems are often at higher risk of developing co-infections caused by bacteria and fungi (Franquet, 2004). However, a study of autopsies revealed that 50% of 10 immunocompetent IA patients had respiratory co-infections, of whom 6 had COPD (Mudrakola et al., 2022). Bacterial infection is common in COPD-IPA patients. However, research on supportive biomarkers in COPD-IPA patients with bacterial infection remains limited. CRP and PCT are biomarkers commonly used to detect bacterial infections in their early stages. Paran et al. found that the mean CRP level was 63.77mg/L after analysis of 108 emergency patients with bacterial infection (Paran et al., 2009). In patients with severe bacterial pneumonia, CRP levels can exceed 200mg/L (Karhu et al., 2019). In patients with bacterial pneumonia, the early levels of PCT can reach 0.68-2.5 ng/ml (Self et al., 2017; Ayala-Lopez et al., 2021). CRP, PCT levels were significantly higher in bacterial infections compared to the IPA group in our study. In bacterial-*Aspergillus* co-infection, serum levels of CRP and PCT may be higher in patients than in *Aspergillus* infection alone. ESR and LDH are not highly specific for bacterial infection. In previous studies of patients with bacterial pneumonia, the levels of these biomarkers were not very different from the levels of ESR and LDH in the IPA patients in our study (Kerttula et al., 1987; Quist and Hill, 1995; Huang et al., 2018). The role of CER in bacterial pneumonia remains unattended. Indeed, elevated copper concentrations in the lung have been observed to play an important role in resistance to several pulmonary bacterial infections (Wolschendorf et al., 2011; Johnson et al., 2015). As the most prominent carriers of copper, CER are tasked with increasing lung copper concentrations during bacterial infections. As with the function of copper in fungal infections, high levels of copper in the lungs are toxic to bacteria. Moreover, bacteria are much less tolerant to copper than fungi (Besold et al., 2016). It remains unclear whether the body adjusts the serum concentration of CER according to the different tolerance of bacteria and fungi to copper. In other words, it is still unknown whether there are differences in serum CER concentrations between bacterial and fungal infections. Overall, bacterial-fungal co-infection remains a complex issue. Compared with patients with COPD-IPA alone, we believe that patients with COPD-IPA combined with bacterial infection may have higher levels of CRP and PCT, while ESR and LDH levels do not change significantly and CER levels are uncertain. Of course, the actual levels of these biomarkers in patients with COPD-IPA combined with bacterial infection need to be further investigated in the future to meet actual clinical needs.

Our study has several limitations. Firstly, it was difficult to analyse the biomarkers in the COPD-IPA patients with bacterial infection due to limited sample size. Studying supportive biomarkers of IPA in COPD patients merely is difficult to fully meet realistic needs. Supportive biomarker studies in *Aspergillus*-bacterial co-infection need to be complemented in the future. In our study, the control patients all had stable COPD and we did not perform invasive respiratory sampling (e.g. BALF) on them because they did not show signs of acute exacerbation and their lung function status was poor. It was therefore difficult to completely exclude interference from potential pathogens. This may lead to some under-estimation of the ROC curve area for each biomarker. As mentioned above, patients with GOLD stage III and IV COPD have poor lung function, making extensive invasive biopsies difficult. As in most studies of IPA, the IPA population in our study was “probable IPA”. Future studies should include more patients with biopsy-proven IPA. In addition, information on antibiotic use prior to diagnosis of IPA is incomplete, which may lead to false positives for some biomarkers. These issues need to be considered in future studies. All biomarkers were measured on the first day of hospitalization, which prevented us from assessing changes in each biomarker throughout the hospitalization. Therefore, the prognostic stratification for IPA in this study can only be used for the initial assessment of patients. Specific changes in a patient’s condition would still need to be based on real-time changes in biomarkers. In addition, the extent to which dynamic changes in these biomarkers reflect the severity of disease in patients with COPD-IPA and whether they can guide the use of clinical antifungal medications needs to be explored in future follow-up cohort studies. Finally, information on patients’ clinical manifestations was partially lacking. More multicenter prospective cohort studies are needed in the future to investigate COPD-IPA patients with bacterial co-infections and the dynamics of serum supportive biomarkers.

## Conclusions

Our study demonstrated the diagnostic value of CRP, ESR, PCT, LDH, and CER for COPD-associated IPA. However, the combination of these two biomarkers does not significantly enhance diagnostic performance. Elevated levels of CRP and LDH can provide some indication of early assisted ventilation, and early elevation of CRP is also somewhat suggestive of a prolonged hospital stay. Of course, real-time changes in biomarkers remain the more important basis. Clinicians should closely monitor these serum biomarker levels and tailor treatment strategies to improve patient prognosis. Furthermore, we introduce CER as a novel diagnostic marker for IPA patients, highlighting the need for further research to elucidate its role.

## Data availability statement

The original contributions presented in the study are included in the article/**Supplementary Material**. Further inquiries can be directed to the corresponding authors.

## Ethics statement

The studies involving humans were approved by the ethics committee of the First Affiliated Hospital of Guangzhou Medical University (Ethical number: 2018-119). The studies were conducted in accordance with the local legislation and institutional requirements. The human samples used in this study were acquired from primarily isolated as part of your previous study for which ethical approval was obtained. Written informed consent for participation was not required from the participants or the participants’ legal guardians/next of kin in accordance with the national legislation and institutional requirements.

## Author contributions

ZWL: Conceptualization, Writing – original draft, Writing – review & editing. XTL: Investigation, Writing – original draft, Writing – review & editing. ZTL: Conceptualization, Investigation, Writing – original draft. JYH: Formal analysis, Writing – review & editing. YQ: Investigation, Writing – review & editing. SQL: Investigation, Writing – review & editing. YQZ: Investigation, Writing – review & editing. FY: Conceptualization, Project administration, Writing – original draft. YW: Conceptualization, Project administration, Supervision, Writing – original draft, Writing – review & editing.
