# Supplementary material for: Improved diagnostic markers for invasive pulmonary aspergillosis in COPD patients
Source: Front Cell Infect Microbiol. 2024 Apr 3;14:1294971. doi: 10.3389/fcimb.2024.1294971 (PMC11021593; doi:10.3389/fcimb.2024.1294971)
Supplement: Supplementary file 1 [file DataSheet_1.docx]

Supplementary Material

**
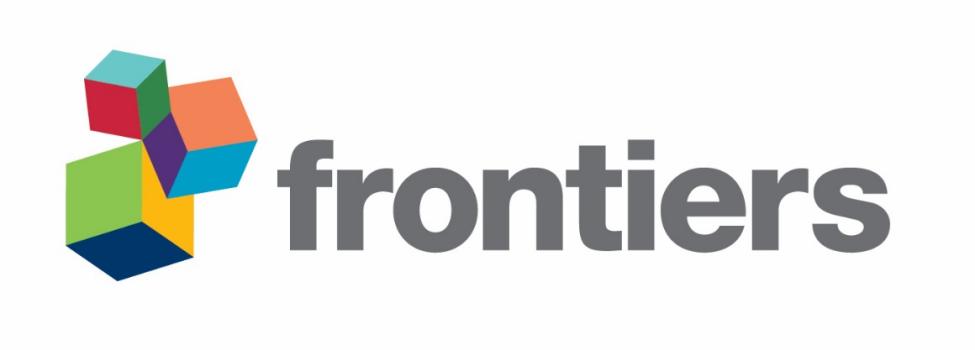
**

**Supplementary Figure 1.** Receiver operating characteristic curve(ROC curve) for 2 biomarkers combined in the IPA and non-IPA groups(Those with a smaller Area under the curve than a single biomarker)

**(A)**
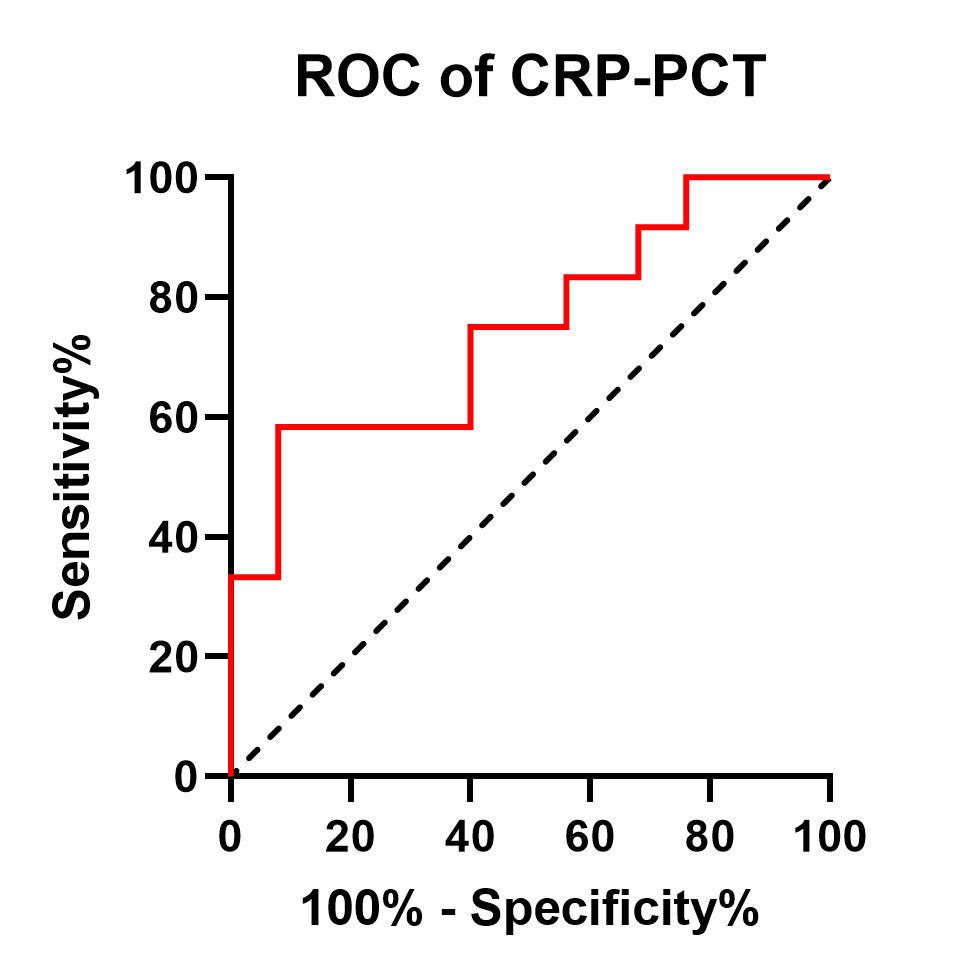
**(B)**
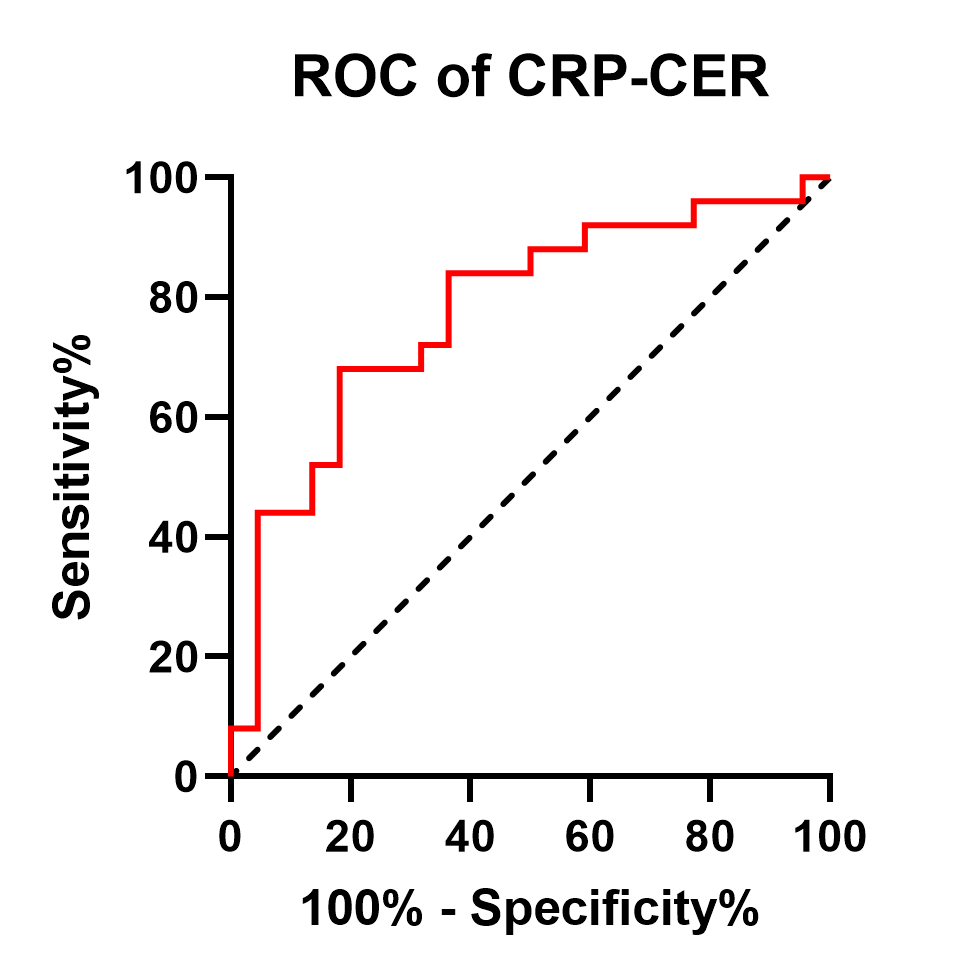


**(C)**
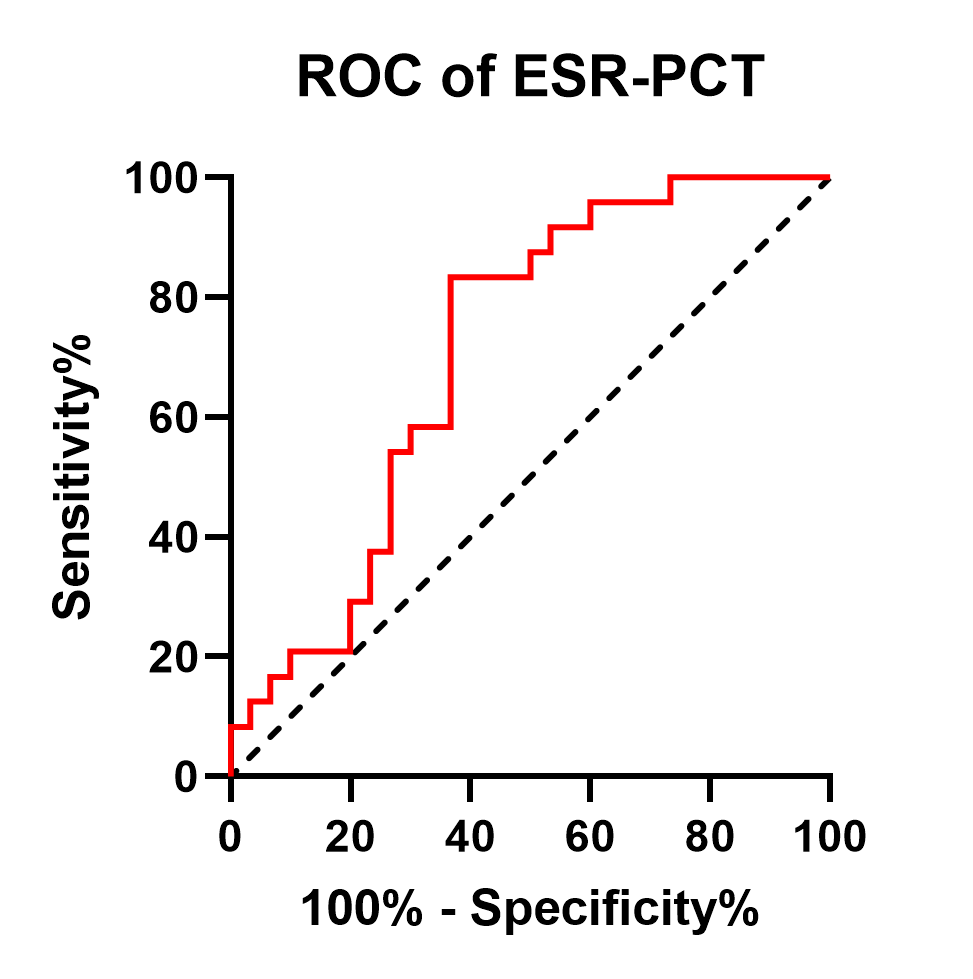
**(D)**
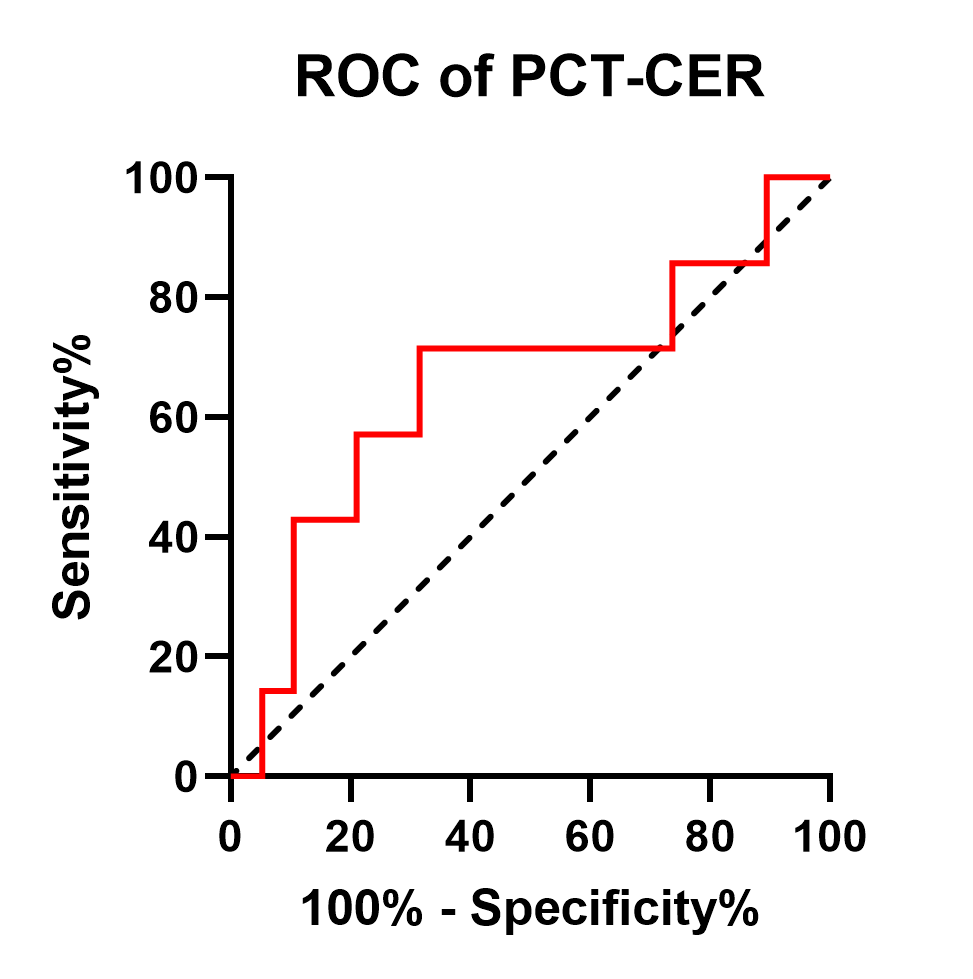


**(E)**
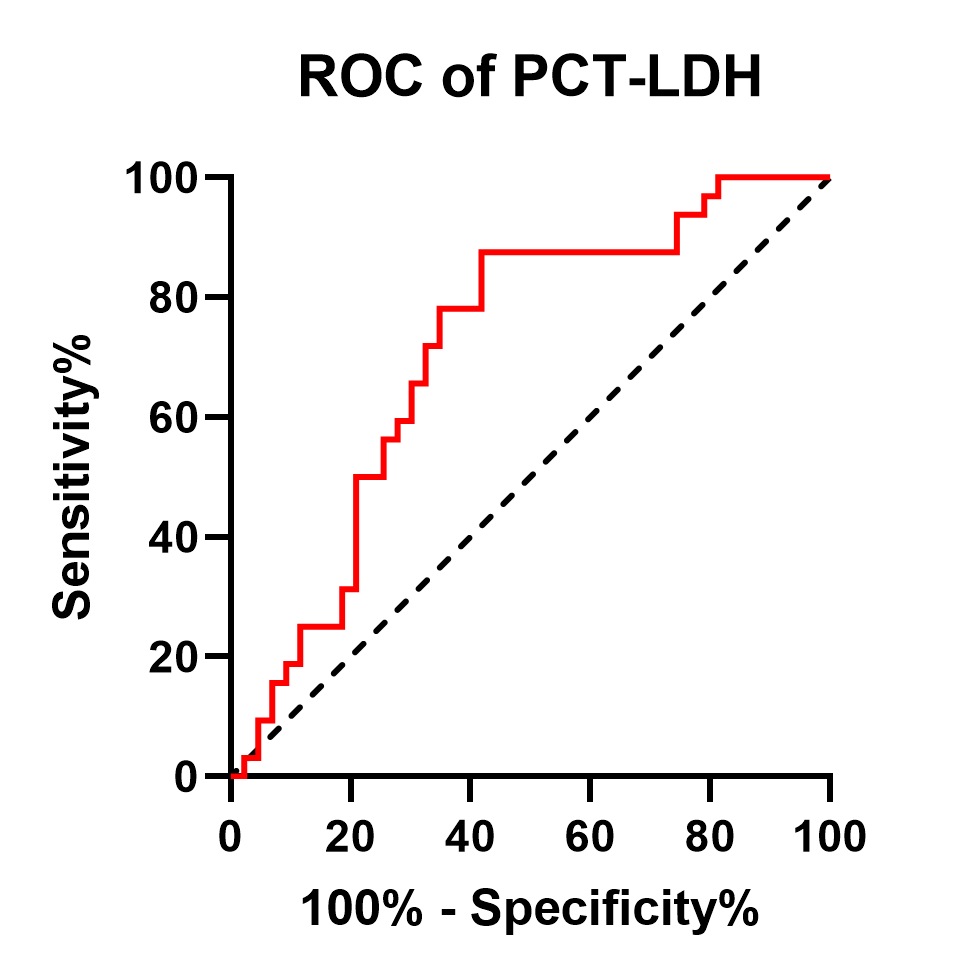


**Supplementary Table 1.** Bulpa criteria

| Proven invasive pulmonary aspergillosis   1. Histopathology or cytopathology showing evidence of Aspergillus hypha and associated tissue damage. 2. One of following：   i)Positive culture of Aspergillus spp. from any LRT sample.  ii)Positive serum antibody/antigen test for A. fumigatus (including precipitins). iii)Confirmation that the hyphae observed are those of Aspergillus by a direct molecular, immunological method and/or culture.  Probable invasive pulmonary aspergillosis   1. COPD patients with GOLD stage III or IV.   2. Treated with steroids.  3. Recent exacerbation of dyspnoea.^a^  4. Suggestive chest imaging^b^ (radiograph or CT scan; <3 months)  5. One of the following:  i)Positive culture and/or microscopy for Aspergillus from LRT.  ii)Positive serum antibody test for A. fumigatus (including precipitins).  iii)Two consecutive positive serum galactomannan tests.  Possible invasive pulmonary aspergillosis   1. COPD patients with GOLD stage III or IV. 2. Treated with steroids.   3. Recent exacerbation of dyspnoea.^a^  4. Suggestive chest imaging^b^(radiograph or CT scan; <3 months)  5. Without positive Aspergillus culture or microscopy from LRT or serology.  Colonization   1. Positive Aspergillus culture from LRT 2. Without exacerbation of dyspnoea, bronchospasm, or new pulmonary   infiltrate. |
| --- |

LRT, lower respiratory tract;COPD, chronic obstructive pulmonary disease;GOLD, Global Initiative for Chronic Obstructive Lung Disease;CT, computed tomography

^a^Exacerbation of dyspnoea and/or bronchospasm resistant to appropriate treatment including antibiotics.

^b^Pulmonary lesions do not respond to appropriate antibiotics.
